# Strategies to enhance the production of pinoresinol and its glucosides by endophytic fungus (*Phomopsis* sp. XP-8) isolated from Tu-chung bark

**DOI:** 10.1186/s13568-018-0584-5

**Published:** 2018-04-16

**Authors:** Jing Zhu, Lu Yan, Xiaoguang Xu, Yan Zhang, Junling Shi, Chunmei Jiang, Dongyan Shao

**Affiliations:** 1 School of Food Sciences, Xinyang Agriculture and Forestry University, 1 North Perimeter Road, Xinyang, 464000 Henan China; 2 Key Laboratory for Space Bioscience and Biotechnology, School of Life Sciences, Northwestern Polytechnical University, 127 Youyi West Road, Xi’an, 710072 Shaanxi China; 3 College of Food, Shihezi University, Road Beisi, Shihezi, 832003 Xinjiang China

**Keywords:** *Phomopsis* sp. XP-8, Lignans, RT-qPCR, Co-culture

## Abstract

To improve the production yield of (+)-pinoresinol (Pin), (+)-pinoresinol monoglucoside (PMG), and (+)-pinoresinol diglucoside (PDG), different methods were conducted, including co-culture with resveratrol-producing *Alternaria* sp. MG1 spores and addition of Tu-chung in a medium at the start of cultivation, ultrasound treatment (40 kHZ, 10 min) on 5-day culture, and addition of ethanol and sodium butyrate on Day 3, followed by cultivation for an additional period of 2 days. At the end of the cultivation period (5 days), the liquid phase was collected for product analysis. Cells were collected for the determination of gene expression levels and then used in bioconversion using resting cells for another period of 2 days. The liquid phase was measured to determine the output of the target products and the expression levels of the key genes related to the biosynthesis of these compounds. Consequently, co-culture with *Alternaria* MG1 and addition of Tu-chung bark in the medium efficiently increased Pin, PMG, and PDG production yield in the biosynthesis systems using potato dextrose broth medium and resting cells of *Phomopsis* sp. XP-8. The key genes related to the biosynthesis of these compounds were significantly upregulated. However, in the majority of cases, the addition of ethanol and sodium butyrate, and ultrasound treatment decreased the production yield of Pin, PMG, and PDG. The change in production yield was not consistently accompanied by a change in gene expression.

## Introduction

Lignan is a type of polyphenol that is widely found in plants. Pinoresinol [(+)-Pin] is a simple lignan converted from 2 coniferyl alcohol molecules via the phenylpropanoid pathway followed by the lignan biosynthesis pathway. Many lignan compounds and their derivatives have been found to have novel biological functions, including antibacterial and anticancer activities. Pin exhibits a considerably stronger anti-inflammatory activity against human intestinal Caco-2 cells, compared with other tested plant lignans (During et al. 2012). (+)-Pin and its glycosylation derivatives, pinoresinol diglucoside ((+)-1-pinoresinol 4,4′-di-*O*-β-d-glucopyranoside, PDG), have been identified as enterolactone precursors with preventive effects against breast cancer (Horn-Ross et al. 2001) and endometrial cancer (Gumma and Ramesh 2003). (+)-Pin has also exerted putative hypoglycemic effects via the inhibition of α-glucosidase (Wikul et al. 2012). PDG has been found to exhibit various pharmacological functions against hypertension (Luo et al. 2010) and osteoporosis (Saleem et al. 2005). After dietary consumption, PDG can be converted to enterolignans by intestinal microflora (Xie et al. 2003). As such, it can potentially reduce the risk of breast cancer (Xie et al. 2013) and other hormone-dependent cancers (Adlercreutz 2002).

The demand for lignans, including PDG and Pin, has rapidly increased as therapeutic materials have been developed in recent years (Satake et al. 2013). Almost all lignans are still mainly produced by extraction from plant materials because of their complex biosynthesis pathway and the involvement of multiple steps and enzymes. However, extraction from plants is not suitable for the scale-up production of these compounds because of the long growth period required for plants and low production yield of these compounds in nature. Microbial fermentation is regarded as having the greatest potential to produce these compounds owing to its high efficiency, easy of regulation, fast bioconversion, and absence of seasonal limitations. The recombination of multiple genes for metabolic remodeling is currently regarded as the most efficient technique in producing original plant products. However, this method is difficult to implement for lignan production because multiple genes and steps are required in the biosynthesis pathway. *Phomopsis* sp. XP-8 is an endophytic fungus isolated from the bark of Tu-chung (*Eucommia ulmoides* Oliv.), a traditional hypotensor in Chinese herb medicine. *Phomopsis* sp. XP-8 can produce Pin, PDG, and (+)-pinoresinol monoglucoside (PMG) in submerged fermentation (Shi et al. 2012), mung bean solid medium, and bioconversion systems with resting cells (Zhang et al. 2015). As such, it shows potential in the production of these compounds without the need for genetic modification.

However, the current yield of Pin and PDG in *Phomopsis* sp. XP-8 is considerably low, failing to satisfy the requirements for scale-up production. In addition, few studies have been successfully conducted on the development of an endophyte as a commercial producer of biological molecules apart from secondary metabolites in plants. Many methods have been conducted to improve the secondary metabolite yield and productivity of endophytes, such as mutagenesis (Zhou et al. 2010), genetic transformations (Liu et al. 2013), optimization of fermentation parameters (Wang et al. 2014), elicitor/inhibitor addition (Venugopalan and Srivastava 2015), precursor feeding (Guerrabubb et al. 2012), use of adsorbent resins/solid supports (Luo et al. 2014; Singh et al. 2010), co-cultivation and mixed fermentation (Kusari et al. 2011), and use of epigenetic modifiers (Brakhage 2013). Co-culture of different organisms, including plant and different microorganisms (Soliman and Raizada 2013; Ola et al. 2013), addition of sodium butyrate (Jeremy et al. 2012) and ethanol (Zhao et al. 2013), and ultrasound (Schläfer et al. 2000) treatment, can improve the production yield of lignans and other products related to the phenylpropanoid pathway.

In the present study, co-culture of *Phomopsis* sp. XP-8 and *Alternaria* sp. MG1, an endophytic fungus isolated from grape that can produce resveratrol via the phenylpropanoid pathway-like pathway, and co-culture of *Phomopsis* sp. XP-8 and Tu-chung bark were employed to enhance the production yield of Pin, PMG, and PDG. Ultrasound treatment and addition of ethanol and sodium butyrate were also conducted. Previously developed liquid fermentation and the bioconversion system with *Phomopsis* sp. XP-8 resting cells was used (Zhang et al. 2015, 2016b). The expression of the key genes (4-coumarate: CoA ligase, 4CL; chalcone synthase, CHS; UDP-glucosyl transferase, GT) related to the biosynthesis of Pin, PMG, and PDG was determined, and the yield of these compounds was monitored accordingly. The present study provides useful information for further research on *Phomopsis* sp. XP-8 and the lignan biosynthesis pathway.

## Materials and methods

### Microorganisms

*Phomopsis* sp. XP-8 (CCTCC M 209291) and *Alternaria* sp. MG1 (CCTCC M 2011348) were used in the study. They were maintained at the China Center for Type Culture Collection (Wuhan, China).

### Preparation of fungal cells

*Phomopsis* sp. XP-8 was prepared as seed cultures, and *Alternaria* sp. MG1 was prepared as spore suspensions for all experiments in the study. The seed cultures of *Phomopsis* sp. XP-8 were prepared in a liquid potato dextrose medium (PDB) by cultivating 5 colonies (5 mm in diameter) formed in a 5-day potato dextrose agar (PDA) culture for 3 days at 28 °C and 180 rpm (Zhang et al. 2016b). *Alternaria* sp. MG1 was prepared as spore suspensions of 1 × 10^6^ spores/mL from the 5-day PDA (Che et al. 2016).

## Methods

**Fig. 1:**
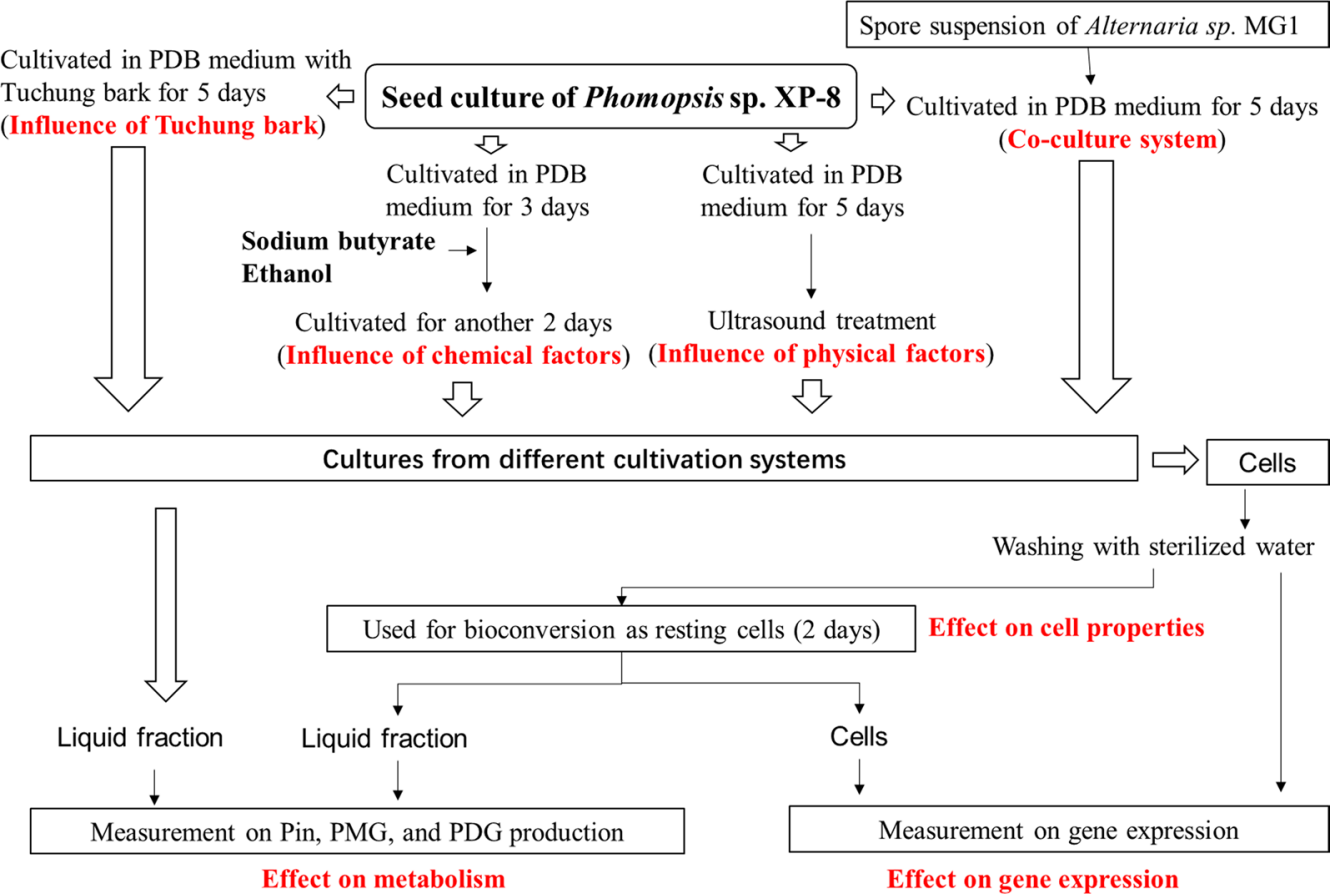
Methods used in the study

Five methods were employed in the study to evaluate their effects on the yield of target products directly after cultivation in a PDB medium and in the bioconversion systems with only glucose and resting cells. The expression levels of the key genes related to the biosynthesis of Pin, PMG, and PDG were also measured to indicate the possible mechanisms underlying these effects. The outline of each approach is indicated in Fig. 1 and detailed below.

In the co-culture method, a system was constructed using a PDB medium (100 mL in a 250 mL flask) with the *Phomopsis* sp. XP-8 seed culture (5% inoculum size), together with the prepared *Alternaria* sp. MG1 spore suspension at the inoculum size of 1%. The culture that was only inoculated with *Phomopsis* sp. XP-8 was also used as the control. After inoculation, cultivation was conducted for 5 days at 28 °C and 180 rpm.

The Tu-chung bark method was employed by cultivating *Phomopsis* sp. XP-8 in the PDB medium with Tu-chung bark added. To prepare the medium, 5 g Tu-chung bark was cut into small pieces (about 0.5 mm) and then added into a PDB medium (100 mL in a 250 mL flask). After sterilization for 20 min at 121 °C, the PDB medium with Tu-chung bark was used to cultivate *Phomopsis* sp. XP-8 at the inoculum size of 5%. The cultivation in PDB without Tu-chung bark was used as the control. The contents of the target products in the PDB medium containing Tu-chung bark but without *Phomopsis* sp. XP-8 was used as the control. All cultivations were conducted for 5 days at 28 °C and 180 rpm.

The method involving the addition of chemicals was applied by cultivating *Phomopsis* sp. XP-8 in a PDB medium (100 mL in a 250 mL flask) at the inoculum size of 5%. After cultivation for 3 days at 28 °C and 180 rpm, sodium butyrate and ethanol were added to the medium at final concentrations of 1 mmol/L and 3% (v/v), respectively. The cultures were continuously cultivated for another period of 2 d. The cultivation in PDB without any chemical addition was used as the control.

For the ultrasound method, the 5-day culture of *Phomopsis* sp. XP-8 was treated with ultrasound for 10 min at 40 kHz. Cultivation was conducted for 5 days in PDB (100 mL in 250 mL flask) at the inoculums size of 5% and under cultivation conditions of 28 °C and 180 rpm. The culture without ultrasound treatment was used as the control.

Subsequently, all cultures from different systems were separated as the liquid fraction and the cells by centrifugation at 5000×*g* for 10 min at 4 °C. The liquid fraction was used to measure the production yield of Pin, PMG, and PDG. A portion of the cells was measure the gene expression levels of the key enzymes for the biosynthesis of these compounds. The residue cells were used as resting cells for bioconversion at the ratio of 10 g wet cells/100 mL bioconversion system (distilled water, pH 7.0, 15 g/L glucose). Bioconversion for 2 days in a 250 mL flask at 28 °C and 180 rpm was conducted. At the end of the bioconversion, the cells were collected by sterile filtration and then used for gene expression analysis. The liquid fraction was also collected to measure the production yield of Pin, PMG, and PDG.

### Analysis of key gene expression

RNA extraction was performed using a commercial kit (Sangon Biotech Co., Ltd. Shanghai, China) in accordance with the protocol provided. The obtained RNA samples were qualitatively and quantitatively measured by using a NanoDrop 2000 spectrophotometer (Thermo Scientific, Waltham, USA) and by gel electrophoresis. cDNA was synthesized from 0.3 μg of total RNA by using the One-Step gDNA Removal and cDNA Synthesis SuperMix kit (Transgen Biotech Co., Ltd. Beijing, China) in accordance with the protocol provided. The cDNA was conserved at − 80 °C prior to use.

Five candidate genes were selected as reference genes from the transcriptome database of *Phomopsis* sp. XP-8 (NCBI code: SRP100582, SRA code: PRJNA376069). These genes included *B*-*TUB1* (encoding α-tubulin), *B*-*TUB2* (encoding α-tubulin), *Rps*-*24* (encoding ribosomal protein S24), *UBC* (encoding ubiquitin-conjugating enzymes), and *α*-*ACTIN* (encoding α-actin). The internal reference genes were the same as those previously reported. All primers were designed using Primer 5.0 (http://www.premierbiosoft.com/primerdesign/index.html) with an amplicon length ranging from 100 bp to 300 bp. Primer specificity was evaluated in silico by BLAST analysis and agarose gel electrophoresis. Amplification efficiency was calculated by dilution. The genes, primers, and efficiencies are listed in Table 1. The measurement of the expression levels of different candidate reference genes was conducted in the system containing *Phomopsis* sp. XP-8 cells in the PDB medium on Days 2, 3, 4, and 5.

**Table 1:** Primers used in gene expression analysis

Genes	Forward primer	Reverse primer
UBC	CGT CGG AAC GAA TCA CAG TA	GCC ACC TAA ACG CAT ACC TC
β-TUB1	GGG AAC GAG GAG GTG AAT AA	GGA TGC TGT CTG AAC TGG AG
β-TUB2	TCG TGT TCG GAG ATA TGC AG	GAC GCG GTT GTA GTG TTT GA
Rps24	AAG CAA CGC AAG AAC CGT AT	CTA TCA ACG CCC AGT CAT CA
α-Actin	GGT CTT TGT TGG GCG AAT	AAA CCA CAG CAT TGT TCC AC
4CL	GTG CAG CAA CTA CGT TCC ATC CT	GCG ACC TGT AGA CCC TTC ACC TT
GT	CTG CTA AGC CAG GAC GGA AGA GG	GAG TCG GAG GTG AAG TCG GAA GAA
CHS	CGC AGT GGT CCT GAG TAA TG	TCA ACA TCA AAG CCC AAG TC

Real-time PCR was performed in 96-well plates on Bio-Rad iCycler (Bio-Rad) using SYBR Green as the fluorophore. The PCR systems contains 1 μL cDNA, 0.5 μM forward and reverse primer, 10 μL 2× EvaGreen Master Mix (Transgen), and 8.0 μL DNase-free water. Each sample was analyzed twice, and the no-template control for each primer was included in all real-time plates. Amplifications were performed under the following conditions: 95 °C for 2 min, 40 cycles of 95 °C for 15 s, 60 °C for 20 s, and a final extension at 72 °C for 2 min. After comparing different genes (Noti et al. 2015), the best reference gene was selected using GeNorm ver. 3.5 (https://genorm.cmgg.be/) according to the M value after stepwise exclusion (Vandesompele et al. 2002).

The expression levels of the key genes related to the biosynthesis of Pin, PMG, and PDG (*4CL*, *GT*, *CHS*) were examined using the primers listed in Table 1. The gene *UBC* (available in NCBI SRA database with accession number of SRP060338) was used as the reference gene for all measurements because it was relatively stable compared with other tested reference genes. The relative quantification of mRNA expression was performed by normalizing the expression value transformed in relative copy numbers (obtained from Cq values and with efficiency considered).

### Measurement of target products

Pin, PMG, and PDG were simultaneously determined by HPLC equipped with a detector and a Shimadzu Wondasil C18 (250 mm × 4.6 mm) in accordance with a previously described method (Zhang et al. 2015). The temperature of the column was set to 30 °C. The mobile phase consisted of acetonitrile (chromatographic grade; Sigma-Aldrich) (solvent A) and ddH_2_O (solvent B). A multistep gradient was used for all analyses, as follows: 1–10 min, 90% (v/v) B; 10–20 min, 80% B; 20–30 min, 30% B; 30–50 min, 90% B. The flow rate was 1 mL/min, and the sample injection volume was 20 μL. The detection wavelengths were 226, 229, and 227 nm for Pin, PMG, and PDG, respectively. Standard Pin, PMG, and PDG (chromatographic grade; Sigma-Aldrich) were prepared in a methanol solution. The production yield of Pin, PMG, and PDG were calculated with reference to the results of the corresponding standards.

### Statistical analysis

The Tuckey test was performed using the software SPSS (Version 18.0, IBM, Armonk, NY, USA) to evaluate the significance of variation among different treatments. The significance level was set at *p *≤ 0.05. The figures were performed using Origin 8.0 (Origin Software, Inc., OriginLab, USA) software.

## Results

### Determination of suitable reference genes

**Fig. 2:**
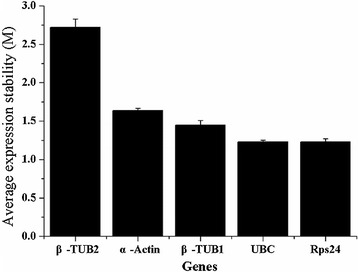
Average expression stability value (M) of candidate reference gene expression in the PDB cultures of *Phomopsis* sp. XP-8

The statistical method GeNorm algorithm was used to identify the M value of different reference genes (Vandesompele et al. 2002). The M value represents the arithmetic average of pairwise variation. After stepwise exclusion, the software excludes the least stable gene (with the highest M value) and recalculates the M value. As shown in the results of GeNorm analysis (Fig. 2), *UBC* and *Rps24* showed the lowest M values, indicating that they were the most stable reference genes used for measurement. *UBC* was more stable than *Rps 24* because the melting curve of *Rps24* was not unimodal in all tests (data not shown).

### Co-culture with *Alternaria* sp. MG1 spores

**Fig. 3:**
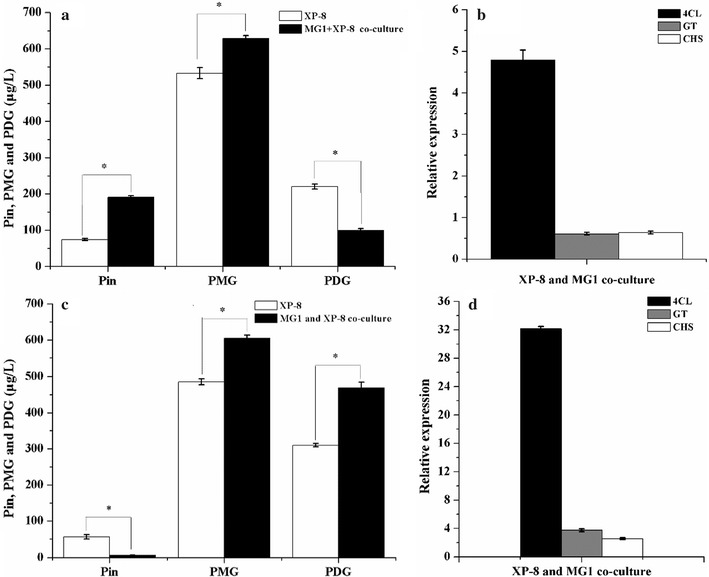
Target product yield and gene expression in the co-culture method with *Alternaria* sp. MG1 spores using PDB medium (**a**, **b**) and the obtained resting cells (**c**, **d**)

Metabolite analyses indicated that co-culture induced a significant increase in Pin production yield by 156.87% (190.60 μg/L) and PMG production yield by 17.89% (628.42 μg/L) but led to a decrease in PDG production yield in bioconversion in the PDB medium by 123% (99.11 μg/L) (Fig. 3a). This result was consistent with the 4.79-fold upregulation in the expression of *4CL* and the 0.61- and 0.64-fold downregulation in the expression levels of *CHS* and *GT*, respectively (Fig. 3b).

In the bioconversion system with resting cells, co-culture with *Alternaria* sp. MG1 spores also showed significant increases in PMG yield by 24.51% (604.69 μg/L) and PDG yield by 51.4% (469.41 μg/L) but exhibited a decrease in Pin yield by 11.62% (6.55 μg/L) (Fig. 3c). This result was consistent with the significant 32.15-, 3.75-, and 2.56-fold upregulation in the expression levels of *4CL*, *GT*, and *CHS*, respectively.

### Addition of Tu-chung bark in the medium

**Fig. 4:**
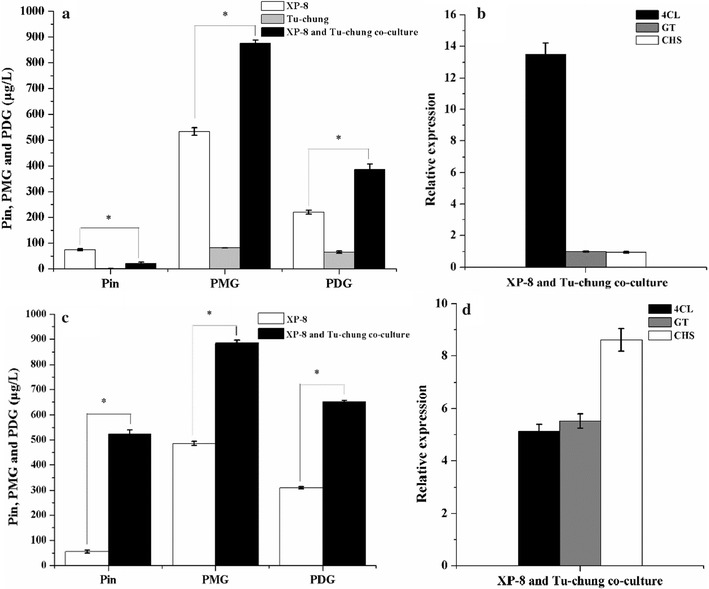
Output levels of target products and expression levels of genes obtained using the method with PDB medium containing Tu-chung (**a**, **b**) and the resting cells (**c**, **d**)

In the system using PDB, addition of Tu-chung bark in the medium increased the PDG and PMG production yield 1.75- and 1.64-fold, respectively, which corresponded to yields amounting to 385.98 and 875.51 μg/L. However, the production of Pin was reduced (Fig. 4a). Correspondingly, the expression of 4CL was upregulated 13.5-fold, whereas the expression levels of GT and CHS were slightly downregulated 0.50- and 0.96-fold, respectively (Fig. 4b).

In the bioconversion systems using resting cells, the cells prepared in the medium containing Tu-chung bark led to significant increases in Pin, PMG, and PDG production yield by 829.48, 88.18, and 110.06%, respectively; these increases correspond to yields amounting to 524.04, 884.76, and 651.27 μg/L (Fig. 4c). Corresponding to these results, the presence of Tu-chung bark in the medium resulted in the upregulated expression of the 4CL, GT, and CHS genes by 5.14-, 5.53-, and 8.61-, respectively, in the resting cells (Fig. 4d).

### Ultrasound induction

**Fig. 5:**
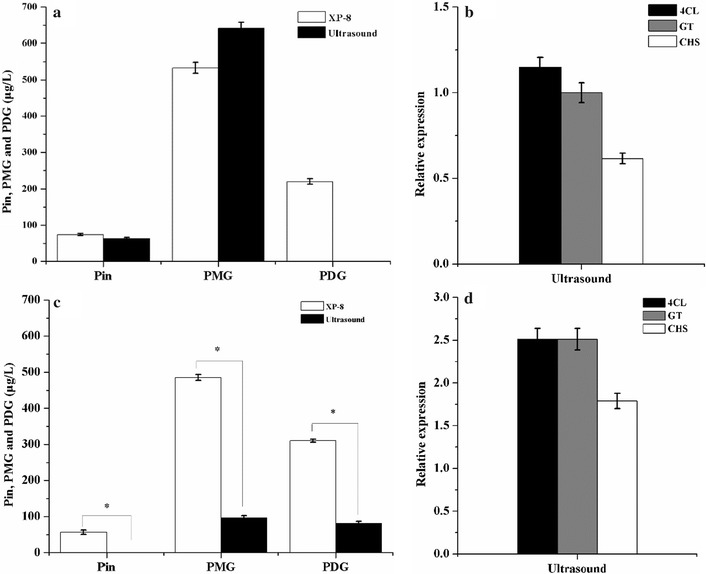
Output of target products and gene expression levels obtained using ultrasound treatment in the systems with PDB medium (**a**, **b**) and the treated resting cells (**c**, **d**)

In the PDB medium, the ultrasound treatment induced a 1.20-fold increase in PMG production yield, corresponding to 640.82 μg/L. However, the production yield of Pin was reduced by 14.91%, and PDG accumulation was not detected in the ultrasound-treated systems (Fig. 5a). Accordingly, the ultrasound treatment resulted in slight changes in the expression of 4CL and GT by 1.14- and 1.15-fold, respectively, but caused a 0.62-fold decrease in the expression of CHS (Fig. 5b).

In the bioconversion system using the resting cells prepared after ultrasound treatment, the production yield of Pin, PMG, and PDG was significantly decreased by 0, 19.91, and 26.18%, respectively, relative to that using the resting cells prepared from the culture without ultrasound treatment. In addition, the ultrasound treatment led to the reduction in resting cells by half. (Fig. 5c). However, the upregulation in the expression levels of 4CL, GT, and CHS relative to the expression of UBC, corresponding to 2.51-, 2.50-, and 1.79-fold increases, respectively, was also induced by ultrasound treatment (Fig. 5d).

### Ethanol induction

**Fig. 6:**
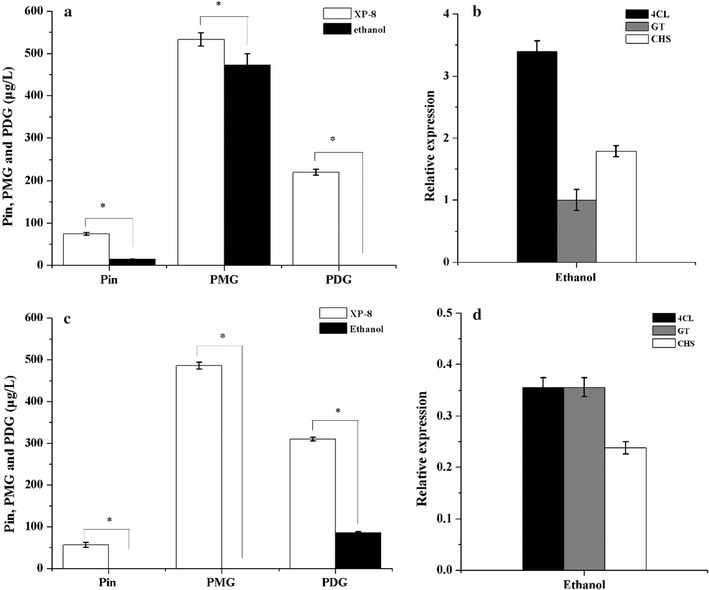
Output levels of target products and expression levels of the genes obtained using ethanol induction in the systems containing the PDB medium (**a**, **b**) and resting cells (**c**, **d**)

In the biosynthesis systems using PDB medium, ethanol induction on day 3 reduced the production yield of Pin, PMG, and PDG by 81.04, 11.44, and 100%, respectively (Fig. 6a). However, 3.39-, 3.39-, and 1.79-fold increases in the expression levels of 4CL, GT, and CHS respectively, were found in the systems using ethanol induction (Fig. 6b). The results for the target products were not consistent with the results for the key genes. This inconsistency indicated that the presence of ethanol caused more significant downregulation in the expression levels of genes related to metabolism than those of the tested genes because UBC encodes the ubiquitin-conjugating enzyme.

In bioconversion systems using the resting cells obtained from the culture treated with ethanol, the output levels of Pin, PMG, and PDG were significantly reduced by 100, 100, and 72.62%, respectively (Fig. 6c). In accordance with these results, the expression levels of 4CL, GT, and CHS genes were significantly downregulated by 65–75% relative to the expression of the UBC gene (Fig. 6d).

Overall, compared with the liquid fraction of the co-culture systems in PDB, the systems using resting cells showed a relatively higher upregulation in *4CL*, *GT*, and *CHS* and a considerably greater production yield in PDG, indicating that the biosynthesis of PDG was highly related to the upregulation in the expression levels of genes *GT* and *CHS*.

### Induction with sodium butyrate

**Fig. 7:**
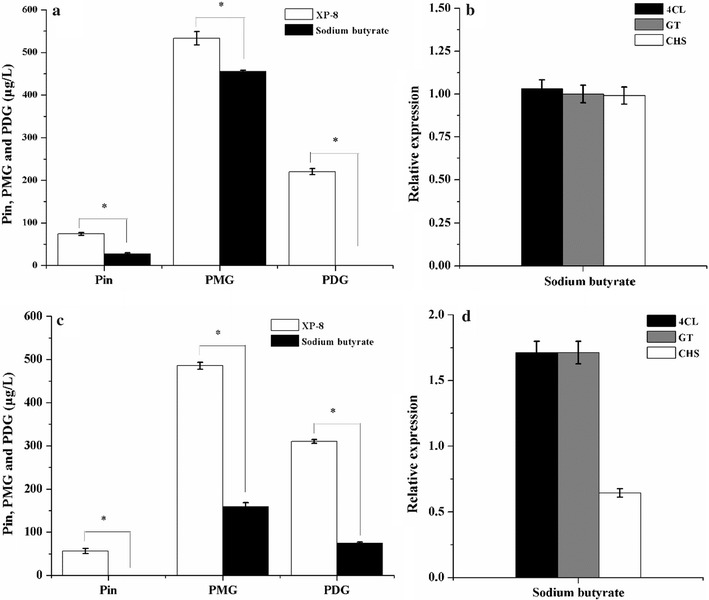
Output levels of target products and expression levels of the genes obtained by sodium butyrate induction in the systems containing PDB (**a**, **b**) and resting cells (**c**, **d**)

In the biosynthesis systems containing PDB, induction with sodium butyrate decreased the output levels of Pin, PMG, and PDG by 63.98, 14.61, and 100%, respectively, which correspond to 26.73, 455.12, and 0 μg/L (Fig. 7a). However, the expression levels of the key genes were slightly affected relative to the control without induction treatment (Fig. 7b).

In the bioconversion systems using resting cells, induction with sodium butyrate reduced the output levels of Pin, PMG, and PDG by 0, 32.68, and 23.81%, respectively (Fig. 7c). However, the expression levels of *4CL* and *GT* remained upregulated 1.71- and 1.71-fold, respectively, relative to the expression of UBC although the expression of CHS was downregulated 0.64-fold when sodium butyrate was used in the induction (Fig. 7d).

## Discussion

### Selection of reference genes for qRT–PCR analysis of *Phomopsis* sp. XP-8

Gene expression profiling has become increasingly important in examining biological systems, particularly in elucidating complex signaling and metabolic pathways underlying developmental, biological, and cellular processes (Yeap et al. 2014). Among the widely used methods of measuring gene expression levels, quantitative real-time PCR (qRT–PCR) is a robust method for assessing mRNA levels across different samples, with the advantages of accuracy, sensitivity, specificity, ability to quantify, and reproducibility (Schmidt and Delaney 2010). The variations are minimized by normalizing gene expression to the expression of one or more reference genes (Bustin et al. 2009). However, the use of inadequate reference genes may lead to errors in interpretation and misinterpretation of data on expression levels (Amil-Ruiz et al. 2013). Thus, appropriate reference genes are a prerequisite for qRT–PCR (Dankai et al. 2015). The stable reference genes in *Phomopsis *sp. XP-8 were first examined in this study although some traditional reference genes have been used for qRT–PCR data normalization in other studies on *Phomopsis liquidambari* (Xie and Dai 2015). In the current study, among the 5 traditional reference genes (*B*-*TUB1*, *B*-*TUB2*, *Rps*-*24*, *UBC*, and *α*-*ACTIN*) tested, *UBC* was identified as a stable expressed gene (Cho et al. 2014); however, multiple reference genes have been reported in other studies (Kong et al. 2015). This finding can facilitate further studies on the exploration of key genes related to secondary metabolites from *Phomopsis* sp. XP-8.

### Factors affecting the output of target products

In the current study, many factors exerted influence on the output of target products. The presence of Tu-chung (the host plant of *Phomopsis* sp. XP-8) and *Alternaria* sp. MG1 (a resveratrol-producing endophytic fungus from grape) can increase the yield of target products, which is in accordance with the upregulation in the expression of key genes. Comparatively, the presence of Tu-chung bark led to the most significant increases in Pin, PMG, and PDG output, particularly in the biosynthesis system with resting cells. This result indicates the promotion of Tu-chung on the secondary metabolites from *Phomopsis* sp. XP-8. In addition, the increase in output of the target products was also observed in the presence of *Alternaria* sp. spores, indicating that fungal and plant composition may exhibit similar properties. However, the addition of ethanol and sodium butyrate and the ultrasound treatment decreased the production yield of Pin, PMG, and PDG; however, an inconsistent change in gene expression was observed. This occurrence could be attributed to these chemical and physical factors, which could damage the viability of fungi.

In addition, the effect of each factor on the production yield of Pin, PMG, and PDG was not always consistent in the biosynthesis system with PDB medium that with resting cells. In our previous study, resting cell culture was found to improve resveratrol and the production yield of Pin (Zhang et al. 2013, 2016a). In other research, the resting cell culture systems were also developed to produce various compounds and to investigate the factors involved in the biosynthesis of bioactive substances (Marchand et al. 2008; Pandey et al. 2011).

### Effect of physical and chemical factors

#### Ethanol

The role of ethanol as a small-molecule elicitor altering the expression of silent (cryptic) secondary metabolite gene clusters is fully documented (Pettit 2011). Ethanol treatment has been found to increase the production yield of Huperzine A (51.89% increment) by endophytic *Colletotrichum gloeosporioides* strain ES026 (Zhao et al. 2013). Marcoleta et al. (2011) demonstrated that ethanol addition promoted carotenogenesis in the yeast *Xanthophyllomyces dendrorhous* by increasing the expression levels of key genes, whereas glucose exerted a repressive effect.

However, in the current study, biomass production decreased in the presence of ethanol (3% (v/v)) in the PDB medium, indicating that ethanol exhibited cellular toxicity, as evidenced by the complete growth inhibition of *Phomopsis* sp. XP-8. Consequently, the output levels of the target products were reduced relative to that of the control. Therefore, ethanol may be associated with the availability of cell growth and the output of the target products of *Phomopsis* sp. XP-8. The growth of the endophyte *C. gloeosporioides* also decreased beyond a certain initial concentration of ethanol in the medium (> 3% (v/v)) during the production of Huperzine A (Zhao et al. 2013). An increase in ethanol concentration beyond 5% (v/v) led to a decrease in Huperzine A production yield because of the detrimental effect on fungal growth. In many other studies, 1% ethanol is the most favorable for fungal growth (Asthana et al. 1971; Zeuthen et al. 1988). Therefore, a further study on different ethanol concentrations should be conducted.

#### Sodium butyrate

Genomic analysis has demonstrated in recent years that some fungi possess essential gene clusters for the production of previously unobserved secondary metabolites. These genes are normally reduced or silenced under most of the conditions. Induction of these genes under stress or special conditions can improve the titers of known compounds or the production of new compounds. Epigenetic modifications in fungi have been used recently to produce new bioactive compounds (Wang et al. 2010; Scherlach and Hertweck 2009). Sodium butyrate has shown to efficiently induce the production of newly found compounds and enhance the production yield of known compounds by inhibiting histone deacetylases (HDAC) in fungi. This finding has been reported in the production of anti-infective cytosporones by the marine endophytic fungus *Leucostoma persoonii* (Shwab et al. 2007). Sodium butyrate was also employed to enhance the production yield of cytosporones B (330%), C (510%), and E (820%) by activating the genes related to biosynthesis of these secondary metabolites (Jeremy et al. 2012). Sodium butyrate was found to competitively inhibit class I HDAC and class II HDAC (Sekhavat et al. 2007). The inhibitory effect of sodium butyrate on *Candida albicans* was also related to the inhibition of chitin synthesis (Braun et al. 1987). About 25–100 mmol/L sodium butyrate inhibited germ tube formation by 40%, whereas 100 mmol/L butyric acid almost completely inhibited germ tube formation (98% inhibition) (Noverr and Huffnagle 2004). In the present study, 1 mmol/L sodium butyrate inhibited the growth of *Phomopsis* XP-8 but did not improve the output levels of Pin, PMG, and PDG. This occurrence could be attributed to the absence of association between HDAC and the biosynthesis of these compounds by using *Phomopsis* sp. XP-8.

#### Ultrasound

Mild intensity ultrasound has been found to affect enzymatic activity, cell membrane and microbial bioconversions (Chen et al. 2008), and secondary metabolite production (Wu and Lin 2002). Ultrasound treatment at intensities lower than 2 W/cm^2^ and frequencies lower than 100 kHz can increase the productivity of biological processes (Liu et al. 2012; Pitt and Ross 2003). Both responses to secondary metabolism elicitation and growth reduction caused by physical elicitors widely vary depending on the dose and time of exposure (Dewir et al. 2010; Kováčik et al. 2009). The optimal conditions for ultrasound exposure were reported as 2–5 min (Rezaei et al. 2011; Wang et al. 2013). A longer exposure period significantly decreased the dry matter, viability, and productivity of target products by using fungi (Sulaiman et al. 2011). However, in the current study, ultrasound exposure (40 kHz) for 10 min caused no significant increase the target products. This finding might be due to the long exposure time, causing damage to fungal cells. Another reason might be that the ultrasound treatment did not disturb the biosynthesis of these compounds. Ultrasound treatment was reported to cause no significant changes in the production yield of total phenolics (Russowski et al. 2013).

#### Co-culture with other fungi

Early co-cultures of different fungal species were conducted in solid-state media in which morphogenesis and metabolic changes were found at the leading edge of the fungal mycelia. The patterns of interaction between the different species were also observed in these co-culture systems. Co-cultures in solid-state media were employed between different fungi, bacteria, and protists (Chakraborty et al. 2017). In recent years, liquid-state media or mixed fermentation have also been used for the co-culture of different species of microorganisms, such as that observed in the natural processes occurring in wine processing (Alonso-del-Real et al. 2017; Sáez et al. 2010). Co-culture of different bacteria, bacteria and fungi, and different fungi have shown great advantages in improving the manufacture of pharmaceutical products because they possess a similar pathway but complementary key genes for the biosynthesis of medicines (Bertrand et al. 2014; Beau et al. 2012).

Co-culture of the endophytic fungus *Paraconiothyrium* SSM001 with a bark fungus (*Alternaria*) caused a 3-fold increase in Taxol production yield (Soliman and Raizada 2013). When SSM001 was pyramided with both the *Alternaria* endophyte along and another fungus (*Phomopsis*) observed to inhabit Taxus, was an 8-fold increase in fungal Taxol production yield from SSM001. Co-culture also induced an increase in the secondary metabolite production in the biosynthesis of the following: subenniatins A and B (Wang et al. 2013); fusaristatin A (Ola et al. 2013); enniatins A1, B1, and B (cyclic depsipeptides) and fusaristatin A–lipopeptide (Ola et al. 2013); podophyllotoxin (Baldi et al. 2010); stemphyperylenol (antifungal polyketide) (Chagas et al. 2013); and 13-oxo-9,11-octadecadienoic acid (oxylipin). In the present study, co-culture of *Alternaria* MG1 and *Phomopsis* XP-8 showed 2.57- and 1.18-fold increases in Pin and PMG production yield in the PDB medium and 1.25- and 1.52-fold increases in Pin and PMG production yield in resting cells. Mechanically, microbial communication may induce the expression of silent lignan gene clusters and thus lead to the production of Pin, PMG, and PDG.

In conclusion, co-culture with *Alternaria* MG1 and the addition of Tu-chung bark in the medium presented an effective method of increasing Pin, PMG, and PDG production yield in a biosynthesis system using PDB medium and that using the resting cells of *Phomopsis* sp. XP-8. Correspondingly, the key genes related to the biosynthesis of these compounds, especially *4CL*, were significantly upregulated. The study indicated a new method to improve the production yields of Pin, PMG, and PDG by *Alternaria* MG1.

## Abbreviations

Pin: (+)-pinoresinol
PMG: (+)-pinoresinol monoglucoside
PDG: (+)-pinoresinol diglucoside
4CL: 4-coumarate: CoA ligase
CHS: chalcone synthase
GT: UDP-glucosyl transferase
PDB: liquid potato dextrose medium
PDA: potato dextrose agar medium
Tu-chung: *Eucommia ulmoides* Oliv
*B*-*TUB1*: the gene encoding α-tubulin
*B*-*TUB2*: the gene encoding α-tubulin
*Rps*-*24*: the gene encoding ribosomal protein S24
*UBC*: the gene encoding ubiquitin-conjugating enzymes
*α*-*ACTIN*: the gene encoding α-actin
BLAST: basic local alignment search tool
PCR: the polymerase chain reaction
cDNA: complementary DNA
SRA: sequence read archive
NCBI: National Center for Biotechnology Information
mRNA: message RNA
HPLC: high performance liquid chromatography
